# The Prognostic Value of PLR in Lung Cancer, a Meta-analysis Based on Results from a Large Consecutive Cohort

**DOI:** 10.1038/srep34823

**Published:** 2016-10-05

**Authors:** Nan Ding, ZhaoFei Pang, Hongchang Shen, Yang Ni, Jiajun Du, Qi Liu

**Affiliations:** 1 Institute of Oncology, Shandong Provincial Hospital Affiliated to Shandong University, Shandong University, Jinan, China; 2 Department of Oncology, Shandong Provincial Hospital Affiliated to Shandong University, Shandong University, Jinan, China; 3 Department of Thoracic Surgery, Shandong Provincial Hospital Affiliated to Shandong University, Shandong University, Jinan, China

## Abstract

Recently, many studies have been conducted to explore prognostic value of platelet to lymphocyte ratio (PLR) for patients with lung cancer, while the results remain controversial. We collected pretreatment, clinicopathological and follow-up data of 1388 lung cancer patients receiving surgery between 2006 and 2011 in our hospital, and reviewed relevant articles from Embase, Pubmed, Web of science databases, then performed a meta-analysis to clarify the relationship between PLR and prognosis of lung cancer patients. Finally, 11 articles with our study were included, results indicated elevated PLR was negatively related to overall survival (HR = 1.33, 95% CI: 1.10–1.62), but not related to progress-free survival (HR = 1.21, 95% CI: 0.97–1.49). Subgroup analysis suggested high PLR was correlated with poor survival in non-small cell lung cancer (HR = 1.43, 95% CI: 1.14–1.78), but not in small cell lung cancer (HR = 1.10, 95% CI: 0.76–1.58). Besides, for patients treated by chemotherapy or radiotherapy (HR = 1.66, 95% CI: 1.15–2.38) and patients in late stage (HR = 1.41, 95% CI: 1.19–1.68), PLR had significantly prognostic value. Additionally, the result was significant for patients when cut-off value of PLR was between 150 and 200 (HR = 1.47, 95% CI: 1.18–1.82). In Conclusion, this meta-analysis revealed that elevated PLR was associated with poor prognosis in lung cancer.

Lung cancer, which is divided into small cell lung cancer (SCLC) and non-small cell lung cancer (NSCLC) for the purpose of treatment, is the most common reason accounting for cancer-related death in males and the second leading cause for cancer-related death in females globally^1^. Although great efforts have been made to improve the level of diagnosis and treatment, the prognosis of lung cancer is still unsatisfied yet, with five-year survival rates of 6.3% for SCLC and 18.2% for NSCLC^2^. So it is still necessary and urgent to find prognostic indicators with good sensitivity and specificity, and the easy-to-access and inexpensive ones will be better.

Through unremitting efforts for several decades, a series of prognostic factors for lung cancer have been identified, such as age, sex, weight loss, smoking status, performance status and TNM stage^3^. However, few of these can be widely used in clinical practice to guide treatment and determine prognosis.

In these years, many studies have proved that systemic inflammation and immunology played important roles in the development and progression of various cancers. Tumor microenvironment is composed of mediators and cellular effectors of inflammation which could promote transformation, proliferation and invasion of cancer cells and influence tumor response to comprehensive therapies^4^,^5^. Some cellular components in hematologic system, which can be detected inexpensively and conveniently in clinical settings, could reflect the status of host inflammation, immunity, and hemostasis^6^. Recently, several hematological markers have been reported to have prognostic utility in many cancers, such as C-reactive protein (CRP), albumin, neutrophils, platelets, lymphocytes, Glasgow prognostic score, neutrophil to lymphocyte ratio (NLR), and platelet to lymphocyte ratio (PLR)^7^,^8^.

To our best knowledge, platelet count was positively associated with metastasis of lymph nodes and negatively correlated with overall survival of patients with lung cancer^9^, while reduced lymphocyte suggested poor prognosis in many cancers^10^. Therefore, PLR, as a representative indicator for systemic inflammation calculated by the number of platelet and lymphocyte, has been researched in many institutes to identify its association with survival of lung cancer patients in these years. However, the majority of these studies had relatively limited sample sizes, and the results were not consistent. So we performed a retrospective study of a large consecutive cohort and conducted a meta-analysis aiming to systematically clarify the prognostic value of PLR in lung cancer.

## Results

### Patients characteristics and survival analysis

1388 lung cancer patients were finally enrolled in our clinical study according to inclusion criteria. The mean age was 58.6, with 1015 patients younger than 65 years old and 373 patients older than 65. There were 982 (70.75%) males and 406 (29.25%) females, with 1292 NSCLC patients and 96 SCLC patients.

**Figure 1:**
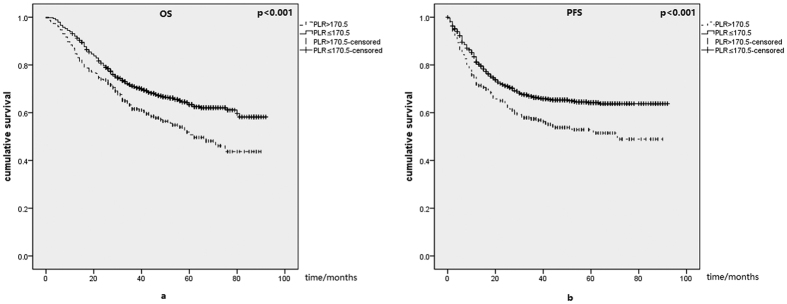
(**a**) Kaplan-Meier curve for PLR and overall survival (OS) of 1388 lung cancer patients. (**b**) Kaplan-Meier curve of PLR for progression free survival (PFS). Patients with elevated PLR had significantly poorer prognosis.

The mean overall survival (OS) were 56.60 for patients with higher PLR and 66.67 for patients with lower PLR. As for mean progression-free survival (PFS), it was 53.78 for patients in PLR ≥ 170.5 group, and it was 64.43 for patients with PLR < 170.5. Kaplan-Meier curves for PLR and OS, PFS were presented in Fig. 1. Patients with elevated PLR had significantly poorer prognosis (p < 0.001). Through univariate analysis, we found that gender, age, pathological type, TNM stage, PLR were significantly related to survival. In Table 1, the results were significant for OS (univariate: 1.451(1.187–1.774); multivariate: 1.405(1.147–1.722)) and PFS (univariate: 1.446(1.183–1.768); multivariate: 1.384(1.130–1.695)) of all patients through both univariate and multivariate analysis. High PLR level was significantly associated with poor OS and PFS in male group. older than 65 group, I/II stage group, NSCLC group, but not in female group, III/IV stage group, SCLC group. As for patients younger than 65 years old, the results were significant for OS and PFS by univariate analysis, while not significant by multivariate analysis.

### Characteristics and selection of studies in meta-analysis

**Figure 2:**
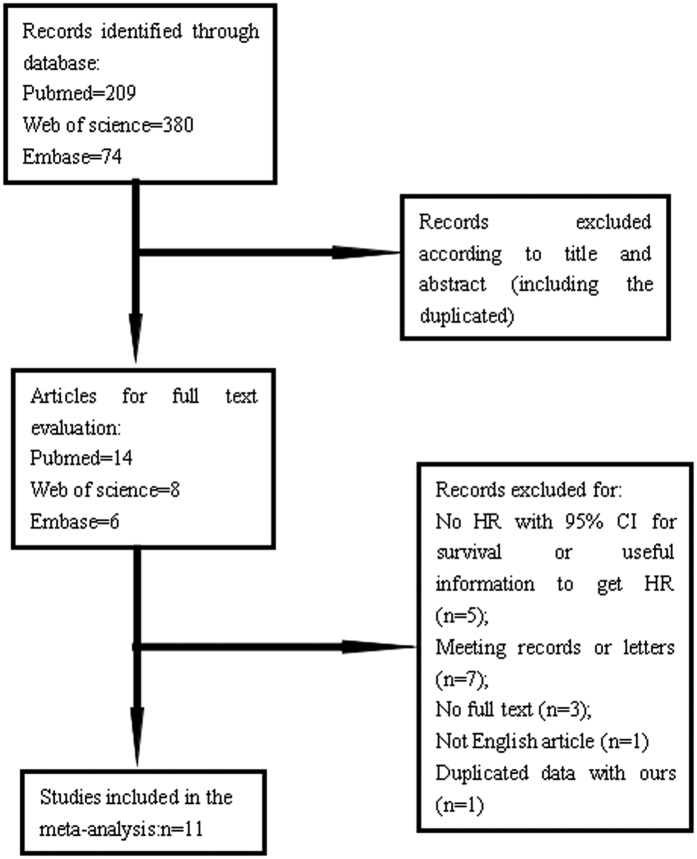
Flow chart to show the process of study selection. Initial searching included 663 studies (209 from Pubmed, 380 from Web of science, 74 from Embase). And through reviewing the abstracts, 28 articles remained. For the further screening of full texts, 11 studies was finally included in our meta-analysis.

Initially, we searched totally 663 studies through Pubmed, Embase and Web of science (Fig. 2) and only 28 records remained through scanning titles and abstracts. In the next round of screening upon full texts, another 16 studies were abandoned. Among these unqualified articles, five of them don’t have HR with 95% CI for survival; six of them are meeting records, while one is a letter; three of them only have conference abstracts without full texts; and one of them was written in Chinese. Finally, we obtained 12 articles, but one study had duplicated data with ours^11^, so 11 articles and our study with 4608 patients were selected for meta-analysis to explore prognostic value of PLR in lung cancer^12^,^13^,^14^,^15^,^16^,^17^,^18^,^19^,^20^,^21^,^22^. Among these included studies, one could be divided into two “sub-studies” for providing sufficient information based on two groups of patients with different stage, we named them as Xie dong1 and Xie dong2, so we thought there were 13 records in our meta-analysis for convenience^22^.

The main information of the 12 eligible articles were shown in Table 2. There were 4608 patients totally with mean age from 57 to 70. 66.2% of them were males, and 33.8% were females. Among them, six were performed in China, two were conducted in Turkey, and the others were studied in Mexico, Republic of Korea, UK, America, and Bulgaria respectively. Eight of them aimed at patients with NSCLC, two were about SCLC, while two contained both NSCLC and SCLC patients. All the studies reported the relationship of lung cancer and OS, only three showed the association between lung cancer and PFS. One study about SCLC included patients with limited and extensive disease together, while the other one had sufficient data for the limited and the extensive separately. As for TNM stage of NSCLC, the patients of three articles were late stage (IIIB–IV/III–IV), one study with early stage (IA–IB), one study for I–III stage disease and IV stage separately. The stage included in the other four articles couldn’t be divided into early or late stage definitely. As we could see, patients from three studies received surgery, while those from five studies underwent chemotherapy or radiotherapy without surgery. The cut-off value of high PLR in these studies ranged from 106 to 300, and the majority were estimated by receiver operator characteristics (ROC) (n = 7). Eight studies included more than 200 patients, while sample number of others were less than 200.

### PLR and OS, PFS in lung cancer

**Figure 3:**
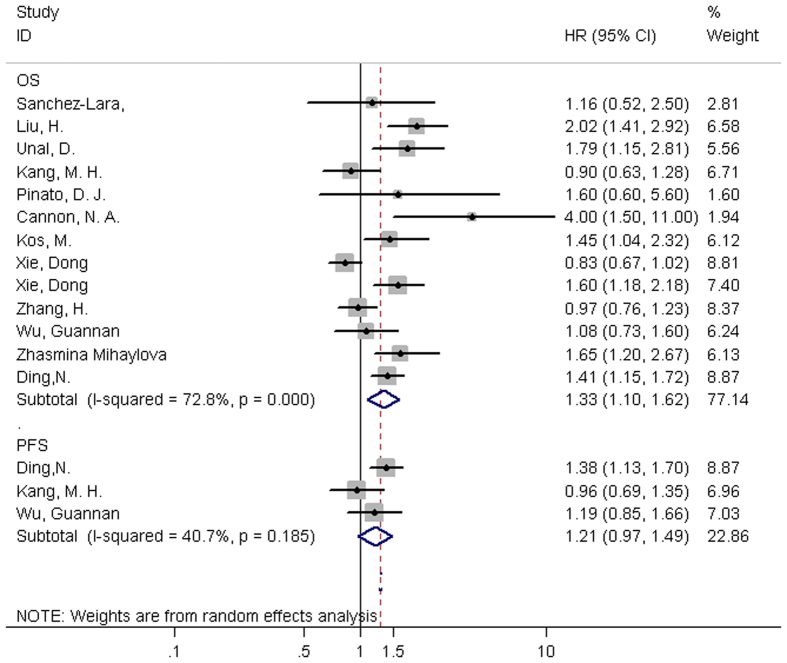
Forest plots of eligible studies evaluating HRs (hazard ratios) of PLR for OS (overall survival), PFS (progress-free survival). P value of Cochran’s Q test and I^2^ value of Higgins I-squared test were also presented.

Sufficient information of PLR and OS in patients with lung cancer was presented in all of the 12 studies. The forest plot showed high PLR was significantly associated with poor OS (HR obtained from random-effects model: 1.33, 95% CI: 1.10–1.62) (Fig. 3). In consideration of the high heterogeneity, we performed subgroup analysis based on type of lung cancer, treatment method, tumor stage, study location, sample size, cut-off value to determine “high PLR”, and methods to estimate HR (Table 3).

Stratification by types of lung cancer, high PLR had significantly prognostic value for patients with NSCLC (HR = 1.43, 95% CI: 1.14–1.78), while it had no significance for patients with SCLC (HR = 1.10, 95% CI: 0.76–1.58). In analyzing treatment method, we found the pooled HRs for patients receiving surgery was 1.20 (95% CI: 0.87–1.65), while 1.66(95% CI: 1.15–2.38) for patients treated by chemotherapy or radiotherapy. As for stage, we could see that the heterogeneity decreased to less than 50% in late stage group, and the combined HRs were 2.17 (95% CI: 0.86–5.49) for early stage group, and 1.41 (95% CI: 1.19–1.68) for late stage group. And high PLR significantly related to poor prognosis for patients in western countries (HR = 1.64, 95% CI: 1.32–2.04), but not for patients in eastern countries (HR = 1.19, 95% CI: 0.93–1.51). We found that there was no heterogeneity for studies from western countries. To analyze cut-off value of “high PLR” on evaluating HR, only the studies with cut off value between 150 to 200 had statistically significant HR 1.47 (95% CI: 1.18–1.82), whereas those whose cut-off value was more than 200 or less than 150 were not significant with pooled HRs of 1.20 (95% CI: 0.69–2.11) and 1.22 (95% CI: 0.82–1.81). And the results were significant both by univariate (HR = 1.53, 95% CI: 1.37–1.72) and multivariate estimate (HR = 1.29, 95% CI: 1.05–1.59).

There were only three studies providing data for PLR and PFS. The forest plot suggested there was no significant relationship between PLR and PFS (HR = 1.21, 95% CI: 0.97–1.49). So it was unnecessary to conduct subgroup analysis for PFS.

### Heterogeneity, Sensitivity analysis, Publication bias

I^2^ for heterogeneity of studies about PLR and OS was 72.8%. And I^2^ was 40.7% for PFS. The meta-regression analysis we performed showed that the treatment method, study location, cancer type, tumor stage, cut-off value of elevated PLR, sample size contributed 12.39%, 13.29%, 13.95%, 7.57%, 14.44%, 12.83% to the source of heterogeneity respectively.

Sensitivity analysis was conducted by removing one study in turn to see if the single study could have significant impact on the pooled HRs for OS. The results were not changed when any study was excluded.

**Figure 4:**
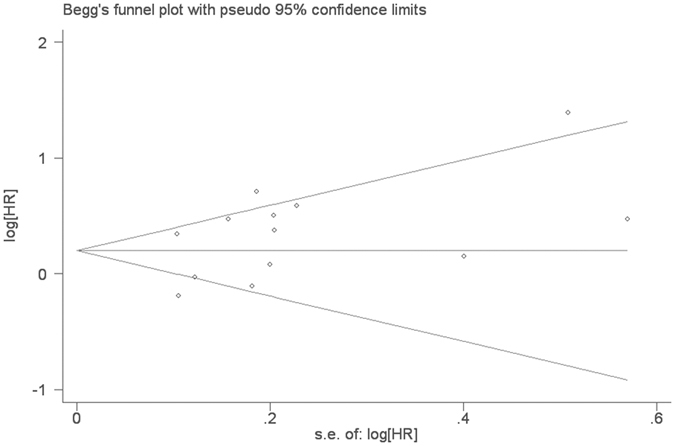
Begg’s funnel plot for analysis of publication bias for OS.

In analysis of PLR and OS, the Begg’s test and Egger’s test for publication bias suggested there was no statistical significance (p = 0.108) (Fig. 4). And there was also no significant publication bias for PFS (p = 0.301).

## Discussion

In the 19^th^ century, Rudolf Virchow found the presence of leukocytes within tumors, firstly indicating of the possible relationship of inflammation and cancer^23^. After efforts of numerous researchers, accumulating evidence suggested that inflammation and immunology played a crucial role in cancer development including tumor genesis, promotion, malignant conversion, invasion, metastasis, and even response to comprehensive therapies and immune defense^24^. An authoritative study mentioned that six biological capabilities composed the hallmarks of cancer^25^, and inflammation played a crucial role. Inflammatory markers and cells interacted with cell matrix to make up tumor microenvironment influencing the occurrence and development of neoplasm^26^. In recent years, some inflammation indicators have been found to be associated with prognosis of patients with various cancers, such as CPR, albumin, neutrophils, platelets, lymphocytes^27^, nutritional index^28^, Glasgow prognostic score^29^, NLR^30^, PI (Prognostic Index), PNI (Prognostic Nutritional Index) and PLR^31^. These parameters have been considered as potential predictors and widely studied mainly because they are cheap, convenient and easy to access in clinician settings. Recently, several studies have been performed to identify the prognostic significance of PLR in lung cancer, however the results were inconsistent. Therefore, we conducted a retrospective study of a large consecutive cohort and performed a meta-analysis aiming to examine the prognostic utility of PLR for survival (OS, PFS) of lung cancer patients.

To our best knowledge, there were two articles of meta-analysis conducting prognostic value of PLR in various cancers. One study did not include the analysis of lung cancer^32^, the other one only included three articles about NSCLC^33^. Zhou, X’s meta-analysis showed elevated PLR was negatively associated with survival in various cancers, such as colorectal cancer, hepatocellular carcinoma, ovarian cancer and NSCLC, but not in gastric cancer and pancreatic carcinoma. Templeton, A.J’s study suggested a significant result was observed for colorectal, hepatocellular, gastro esophageal, ovarian, and pancreatic carcinoma in studies with dichotomized cutoffs of PLR and for colorectal cancers in studies with two cutoffs of PLR. Indeed, only 423 patients of NSCLC were included totally.

Our meta-analysis combined the results of our study and other 11 studies with 4608 lung cancer patients, proving that high PLR had significantly prognostic value for overall survival, but not for progress-free survival. Subgroup analysis indicated the result was significant for NSCLC, not SCLC. More studies should be conducted to explore the prognostic effect of PLR for SCLC, because there were only three articles about SCLC in our analysis. Besides, for patients with NSCLC treated by chemotherapy or radiotherapy, PLR had significant prognostic value for OS. And PLR suggested poor OS when patients were in late stage. These results were relatively credible due to I^2^ value for heterogeneity was less than 50% in this subgroup. Cut-off values of elevated PLR were various, the result was significant when cut-off value of PLR was between 150 and 200. Interestingly, the analysis of studies in western countries showed a significant result with no heterogeneity, and the result was not significant for studies in east with high heterogeneity. However, mechanism of the relationship between PLR and survival in lung cancer patients has not been figured out exactly. Evidence has shown that high platelet count is associated with poor survival of patients with lung cancer^9^. Growth of neoplasm relied on angiogenesis^34^, and some platelet-derived cytokines related to tumor angiogenesis regulatory such as vascular endothelial growth factor (VEGF), basic fibroblast growth factor (bFGF), platelet derived growth factor (PDGF) that have been found elevated in platelets of cancer patients in recent report^35^. On the contrary, lymphocytopenia has been demonstrated to predict a poor prognosis in terms of survival in advanced cancer patients^10^, perhaps due to its effect in mediating tumor cell destruction and inhibiting tumor growth^36^. It has been reported that T cells in the tumor microenvironment might secret cytokines such as interleukin-4 and -5 regulating the proliferation, apoptosis, angiogenesis and metastasis of cancer^37^,^38^.

However, there were some limitations in our meta-analysis. Firstly, the heterogeneity was moderately significant in the pooled HRs of OS (I^2^ = 72.8%, P < 0.001) and mildly significant for PFS (I^2^ = 72%, P = 0.185). The source of heterogeneity might come from complex factors, such as difference of patients (ethnicity, condition, age, sample size, and so on), research method, method to test the number of platelet and lymphocyte, follow-up year, cut-off value of high PLR, statistic method, and so on. Although, we performed subgroup analysis, meta-regression analysis and sensitivity analysis to search the source, none of them could completely explain it. Secondly, we calculated the HR with its 95% CI of one study only providing Kaplan-Meier curve, which might be inaccurate for the final result. Thirdly, there were only three studies with sufficient data for PLR and progress-free survival, lacking of reliability with small sample size. Fourthly, some studies only had univariate analysis for HR, we pooled them with other’s HRs analyzed by multivariate, which might cause some biases. It has to be mentioned that some relevant articles have not been obtained due to the condition limitation.

In conclusion, the meta-analysis suggested that elevated PLR was negatively related to overall survival of patients with lung cancer, especially for NSCLC patients, patients treated by chemotherapy or radiotherapy and patients with PLR cut-off value of elevated PLR between 150 and 200. However, more studies with high quality and large sample size should be conducted to confirm the prognostic value of PLR in lung cancer.

## Methods

### Patients, clinical and follow-up data collection

We collected clinical and pathological characteristics of patients receiving surgical treatment for lung cancer in Shandong Provincial Hospital affiliated to Shandong University between 2006 and 2011, including gender, age, stage, date of surgery, smoking status, platelet count, lymphocyte count, pathological types, and so on. Patients were included if they met the following criteria: 1) diagnosed pathologically as lung cancer; 2) receiving surgical treatment for lung cancer; 3) having pretreatment platelet and lymphocyte count; 4) having integrated follow-up data. The patients who underwent non-cancer related inflammation diseases and other cancers would be excluded.

The follow-up data were collected by phone interview. Overall survival was calculated from date of surgery to death, or the date when patients were out of touch, or cut-off date. Progression free survival was calculated from surgery date to date of progression, or the date when patients were out of touch, or cut-off date.

### Search strategy and study selection and exclusion criteria for meta-analysis

Using key words such as PLR, platelet to lymphocyte ratio, platelet lymphocyte ratio, TLR or thrombocyte lymphocyte ratio, with lung (or pulmonary) cancer, lung (or pulmonary) carcinoma or lung (or pulmonary) tumor, we conducted a literature search via Pubmed, Embase, and Web of Science databases for relevant articles until September, 2015. Both full text and MeSH search for keywords were used. And we used the “related articles” function of Pubmed to broaden our search and reviewed their references to get more eligible articles.

Two researchers (Du, J and Ni, Y) reviewed the eligible articles independently. Once there were disagreements, discussion would be held. Studies were included in the meta-analysis if they met the following criteria: (1) studied patients were diagnosed with lung cancer (both NSCLC and SCLC included) definitely; (2) studies investigating the association of PLR with overall survival (OS), progression-free survival (PFS) or disease-free survival(DFS); (3) Sufficient information reported to estimate the hazard ratio (HR) with 95% confidence interval (CI) of OS, PFS, or DFS; (4) published as a full text in English. Besides, Studies were excluded if they were meeting records, letters, systemic reviews, case reports, or basic laboratory studies.

### Data extraction and Quality assessment

Two investigators (Du, J and Ni, Y) reviewed the title, abstract and full text of the possibly eligible articles to extract data independently, getting the following information: first author, publication year, country of study, number of patients, gender composition, mean age, stage, duration of studies, type of lung cancer, main treatment methods, follow up period, cut-off value of high PLR, methods of survival analysis, HR with 95% CI.

In this meta-analysis, quality assessment was conducted using the Newcastle-Ottawa Scale (NOS), which was designed for retrospective and prospective studies. The Scale includes three parts: selection (0–4 points), comparability (0–2 points), and outcome assessment (0–3 points). The maximum score is 9 points and NOS scores ≥7 are considered as high-quality studies.

### Statistical analysis

The value of PLR was the ratio of platelet count to lymphocyte count. The cut-off point of PLR was decided when the log-rank statistical value was maximum which was identified by receiver operating characteristic (ROC) curve. And 10 repeated times 3-fold internal cross validation was conducted to test the credibility of prognostic value of PLR. Kaplan-Meier (K-M) method was used to determine the significant variables for OS and PFS, and Cox proportional regression method was to test the independence of selected predictors for OS and PFS. These were performed by SPSS (version 20.0) software, and a two-sided p < 0.05 indicated significant.

When performing meta-analysis, we used HR to estimate the relationship of PLR and survival. For some of the eligible articles, we could obtain HR and its 95% confidence intervals directly; for some that only provided Kaplan-Meier curves, we used the methods presented by Parmar^39^ to calculate HR with its 95% CI. Kaplan-Meier curves were read by Engauge Digitizer version 4.1. In some studies, both univariate and multivariate analysis were performed, we chose the latter one for analysis.

The heterogeneity of pooled results was tested by Cochran’s Q test and Higgins I-squared statistic. A p < 0.10 for Q test or I^2^ > 50% for I-squared test revealed there was significant heterogeneity among studies and the random-effects model (DerSimonian-Laird method) was selected^40^. Otherwise, the fixed-effects model (Mantel-Haenszel method) was adopted. To find reasons of heterogeneity among studies, we conducted meta-regression and subgroup analysis using variables as cancer type, HR analysis method, treatment method, stage, cut-off value, sample size and study location. Sensitivity analysis was conducted to test the reliability of outcomes of these studies in our analysis by removing one single study in sequence. Publication bias analysis aimed at estimating the credibility of meta-analysis results, which might be achieved by Begg’s funnel plot and Egger’s linear regression test, and a p < 0.05 was set as significant. In addition, we used the software StataSE12.0 to perform all the statistical analysis.

### Ethical approval

The study acquired the permission by ethic community of Shandong Provincial Hospital afflicted to Shandong University. Informed consent was obtained from all individual participants included in the study. And all the experiments described here were performed in accordance with the approved guidelines.

## Additional Information

**How to cite this article**: Ding, N. *et al*. The Prognostic Value of PLR in Lung Cancer, a Meta-analysis Based on Results from a Large Consecutive Cohort. *Sci. Rep.* **6**, 34823; doi: 10.1038/srep34823 (2016).

## Figures and Tables

**Table 1:** Results of the prognostic value of platelet to lymphocyte ratio (PLR) for the survival of 1388 patients with lung cancer.

	Number of patients	OS				PFS			
		Univariate		Multivariate		Univariate		Multivariate	
		HR	p	HR	p	HR	p	HR	p
**all patients**	1388	1.451(1.187–1.774)	<0.001	1.405(1.147–1.722)	0.001	1.446(1.183–1.768)	<0.001	1.384(1.130–1.695)	0.002
sex									
male	982	1.685(1.346–2.109)	<0.001	1.700(1.353–2.136)	<0.001	1.676(1.339–2.098)	<0.001	1.651(1.314–2.074)	<0.001
female	406	0.894(0.566–1.412)	0.632	0.709(0.446–1.127)	0.146	0.896(0.568–1.414)	0.637	0.728(0.458–1.155)	0.178
age									
>65	373	1.665(1.195–2.321)	0.003	1.635(1.166–2.292)	0.004	1.665(1.195–2.320)	0.003	1.650(1.174–2.318)	0.004
≤65	1015	1.323(1.027–1.704)	0.031	1.280(0.990–1.653)	0.059	1.317(1.022–1.696)	0.033	1.249(0.967–1.613)	0.088
stage									
I/II	933	1.550(1.186–2.024)	0.001	1.502(1.148–1.965)	0.003	1.548(1.185–2.022)	0.001	1.512(1.155–1.979)	0.003
III/IV	455	1.237(0.925–1.655)	0.152	1.318(0.979–1.774)	0.069	1.187(0.887–1.587)	0.248	1.256(0.934–1.688)	0.131
cancer type									
NSCLC	1292	1.462(1.184–1.804)	<0.001	1.393(1.125–1.724)	0.002	1.462(1.185–1.804)	<0.001	1.380(1.115–1.707)	0.003
SCLC	96	1.412(0.719–2.776)	0.317	1.393(0.703–2.758)	0.342	1.343(0.683–2.639)	0.393	1.298(0.655–2.571)	0.454

P < 0.05 is considered to be significant. Abbreviation: HR: hazard ration, NSCLC: non-small cell lung cancer; SCLC: small cell lung cancer, OS: overall survival; PFS: progression free survival.

**Table 2:** The characteristics and main information of included studies.

Author	Years	Country	Sample size	Gender (M/F)	Mean age	Stage	Cancer type	Treatment	Follow up	Cut off value	Survival analysis	HR estimate	Duration
Sánchez-Lara, K.	2012	Mexico	119	55/64	60.5	IIIB/IV	NSCLC	C	6	150	OS	MV	2009.04–2011.02
Liu, H.	2013	China	210	139/71	61	III/IV	NSCLC	C	18.6	152.6	OS	UV/MV	2001.01–2012.08
Unal, D.	2013	Turkey	94	88/6	58.1	II/IIIA/IIIB	NSCLC	C+R	NF	194	OS	UV	NF
Kang, M.	2014	Republicof Korea	187	162/25	68	Limited Extensive	SCLC	C+R	40.28	160	OS/PFS	MV	2006.07–2013.10
Pinato, D.	2014	UK	220	110/110	6 5	IA/IB/IIA/IIB/IIIA	NSCLC	C	NF	300	OS	UV	2004–2012
Cannon, N.A.	2015	America	59	31/28	70	IA/IB	NSCLC	S	17	146	OS	MV	2006.01.01–2012.08.32
Kos, M.	2015	Turkey	145	130/15	57	I/II/III/IV	NSCLC	R	33	198.2	OS	UV/MV	2005–2012
Xie, D.1	2015	China	555	318/237	66.7	Extensive	SCLC	C/R/S/C+S	10.8	210	OS	MV	1997–2012
Xie, D.2	2015	China	383	182/201	66.7	Limited	SCLC	C/R/S/C+S/N	10.8	210	OS	MV	1997–2013
Zhang, H.	2015	China	678	449/229	61	T1 T2 T3-4 N0 N1 N2	NSCLC	C/R/S/C+S/N	43.5	106	OS/DFS	UV/MV	2004.01–2008.13
Wu, G	2015	China	366	246/120	NF	III/IV	NSCLC	S	NF	119.5	OS/PFS	UV/MV	2007.01–2012.09
Mihaylova, Z.	2015	Bulgaria	204	159/45	60.2	IB/IIA/IIB/IIIA/IIIB/IV	NSCLC SCLC	C	NF	188	OS	MV	NF
Ding, N (our data)	2015	China	1388	982/406	58.6	I/II/III/IV	NSCLC SCLC	NF	44	170	OS/PFS	UV/MV	2006.01–2014.07

Abbreviation: NF: not found; C: chemotherapy; S: surgery; R: radiotherapy; N: no treatment; OS: overall survival; DFS: disease-free survival; PFS: progression-free survival; HR: hazard ratio; UV: univariate analysis; MV: multivariate analysis; M: male; F: female; NSCLC: non-small cell lung cancer; SCLC: small cell lung cancer, UK: United Kingdom.

**Table 3:** Results of subgroup analysis about PLR (platelet to lymphocyte ratio) and OS (overall survival) in lung cancer.

Stratified analysis	No. of studies	No. of patients	Random-effects model		Fixed-effects model		I^2^(%)
			HR(95% CI)	p	HR(95% CI)	p	
Histology							
NSCLC	9	3183	1.43(1.14–1.78)	0.002	1.34(1.18–1.51)	<0.001	60.0
SCLC	4	1221	1.10(0.76–1.58)	0.611	1.01(0.87–1.17)	0.904	77.8
Treatment							
Surgery	3	2286	1.20(0.87–1.65)	0.266	1.21(1.04–1.41)	0.016	65.0
C or R	5	848	1.66(1.15–2.38)	0.007	1.61(1.30–2.00)	<0.001	58.4
Tumor stage							
Early stage	2	992	2.17(0.86–5.49)	0.103	1.61(1.24–2.08)	<0.001	71.1
Late stage	5	1204	1.41(1.13–1.76)	0.021	1.41(1.19–1.68)	<0.001	33.5
Country							
Western	6	841	1.64(1.32–2.04)	<0.001	1.64(1.32–2.04)	<0.001	0
Eastern	7	3767	1.19(0.93–1.51)	0.163	1.14(1.03–1.27)	0.009	80.7
Sample size							
≥200	8	4004	1.29(1.02–1.64)	0.034	1.20(1.08–1.33)	<0.001	78.2
<200	5	604	1.45(0.97–2.15)	0.067	1.32(1.06–1.63)	0.012	64.9
Cut–off value							
≤150	4	1222	1.22(0.82–1.81)	0.326	1.06(0.87–1.29)	0.561	59.8
150~200	6	2228	1.47(1.18–1.82)	<0.001	1.44(1.27–1.65)	<0.001	56.8
≥200	3	1158	1.20(0.69–2.11)	0.519	1.03(0.87–1.22)	0.754	84.4
HR estimate							
UV	7	3101	1.53(1.37–1.72)	<0.001	1.53(1.37–1.72)	<0.001	0.5
MV	11	4294	1.29(1.05–1.59)	0.015	1.20(1.09–1.31)	<0.001	75.5

P < 0.05 is considered to be significant.

Abbreviation: PLR: platelet to lymphocyte; OS: overall survival; NSCLC: non-small cell lung cancer; SCLC: small cell lung cancer; UV: univariate analysis; MV: multivariate analysis; UV: univariate analysis; MV: multivariate analysis; HR: hazard ratio; C: chemotherapy; R: radiotherapy.
